# Alginate-Poly(ethylene glycol) Hybrid Microspheres for Primary Cell Microencapsulation

**DOI:** 10.3390/ma7010275

**Published:** 2014-01-09

**Authors:** Redouan Mahou, Raphael P. H. Meier, Léo H. Bühler, Christine Wandrey

**Affiliations:** 1 Institut d’Ingénierie Biologique et Institut des Sciences et Ingénierie Chimiques, Ecole Polytechnique Fédérale de Lausanne, EPFL-SV-IBI-LMRP, Station 15, Lausanne CH-1015, Switzerland; 2 Surgical Research Unit, University of Geneva, CMU-1, Geneva CH-1211, Switzerland; E-Mails: raphael.meier@hcuge.ch (R.P.H.M); leo.buhler@hcuge.ch (L.H.B.)

**Keywords:** alginate, biocompatibility, cell encapsulation, cell transplantation, hydrogel, microencapsulation, poly(ethylene glycol)

## Abstract

The progress of medical therapies, which rely on the transplantation of microencapsulated living cells, depends on the quality of the encapsulating material. Such material has to be biocompatible, and the microencapsulation process must be simple and not harm the cells. Alginate-poly(ethylene glycol) hybrid microspheres (alg-PEG-M) were produced by combining ionotropic gelation of sodium alginate (Na-alg) using calcium ions with covalent crosslinking of vinyl sulfone-terminated multi-arm poly(ethylene glycol) (PEG-VS). In a one-step microsphere formation process, fast ionotropic gelation yields spherical calcium alginate gel beads, which serve as a matrix for simultaneously but slowly occurring covalent cross-linking of the PEG-VS molecules. The feasibility of cell microencapsulation was studied using primary human foreskin fibroblasts (EDX cells) as a model. The use of cell culture media as polymer solvent, gelation bath, and storage medium did not negatively affect the alg-PEG-M properties. Microencapsulated EDX cells maintained their viability and proliferated. This study demonstrates the feasibility of primary cell microencapsulation within the novel microsphere type alg-PEG-M, serves as reference for future therapy development, and confirms the suitability of EDX cells as control model.

## 1. Introduction

Microencapsulation of living cells in spherical hydrogel beads or capsules has been intensely studied for more than three decades. There are many clinical trials going on. Despite enormous accumulation of knowledge documented in abundant original papers, reviews and monographs, which confirm the continuous progress in the field, cell microencapsulation has not yet been translated into any established therapy. While allotransplantation of cells can be performed in combination with immunosuppression, there is consensus that cell xenotransplantation will need immunoprotection of the cells by suitable semi-permeable materials produced by standardized technologies. Both materials and technology have to be well adapted to each specific cell type. Currently, relying on existing knowledge together with research and development on the one hand and focus on the improvement of existing approaches on the other hand, researchers are looking for novel strategies.

Hydrogels composed of either covalently or electrostatically crosslinked biocompatible macromolecules mimic, to a certain extent, the natural environment of cells. The high water content renders hydrogels attractive for cell encapsulation/immobilization intended for subsequent transplantation [1–3].

Alginate-based hydrogels are the most frequently reported materials for cell microencapsulation. Although other natural and synthetic polymers are under investigation, hardly any others have attracted the same attention as the calcium alginate (Ca-alg) or the alginate-poly(L-lysine)-alginate (APA) microcapsule [4]. Despite some known physical limitations, there is no doubt about the advantageous properties of Na-alg and its biological acceptance upon ionotropic gelation with divalent cations. The latter was recently confirmed using a human whole blood model to evaluate the inflammatory properties of several microbeads and microcapsules [5,6]. As evident from recent publications, on the one hand research continues with simple calcium and barium alginate microbeads [7–13], while on the other hand poly(L-lysine) has been replaced by poly(L-ornithine) [14] or chitosan [15–18], or alginate has been combined with other compounds such as carrageenan [19] or synthetic methacrylate-based polymers [20–22]. However, using Na-alg in combination with polycations, the chance of success in terms of biocompatibility is uncertain [23]. Alternatively to Na-alg, agarose [24], sodium cellulose sulfate [25] or poly(ethylene glycol) (PEG) and derivatives of these have been used [26,27]. Further, the modification of the alginate backbone with biomolecules [28] as well as alginate-PEG microspheres obtained by crosslinking via Staudinger ligation have been addressed [29].

Taking into account the advantages of both alginate- and PEG-based hydrogels, we have proposed a novel type of hydrogel microsphere. The combination in a one-step process of the electrostatic interaction of calcium ions with Na-alg and the chemical crosslinking reaction of vinyl sulfone-terminated multi-arm PEG (PEG-VS) yielded alginate-PEG hybrid hydrogel microspheres (alg-PEG-M) with well-controllable physical properties [30]. The physical properties of the alg-PEG-M, which are tunable in a wide range of interesting biomedical applications [31], have motivated us to study the microsphere formation in biologically relevant media and to investigate the feasibility of cell microencapsulation in alg-PEG-M.

This paper reports and discusses cell microencapsulation of primary human foreskin fibroblasts (EDX cells) as a model. The study was designed to answer primarily the following questions: Is the microsphere formation influenced when it is performed in cell culture medium? How does the PEG concentration influence the stability and durability of alg-PEG-M? Is cell microencapsulation feasible in this novel two-component hydrogel prepared in one step without any further coating or reinforcement? Do primary cells maintain their viability for a longer period upon microencapsulation in alg-PEG-M? This is the first study to address these questions for alg-PEG-M.

## 2. Results and Discussion

### 2.1. Formation of Alg-PEG-M in Cell Culture Medium

**Figure 1.**
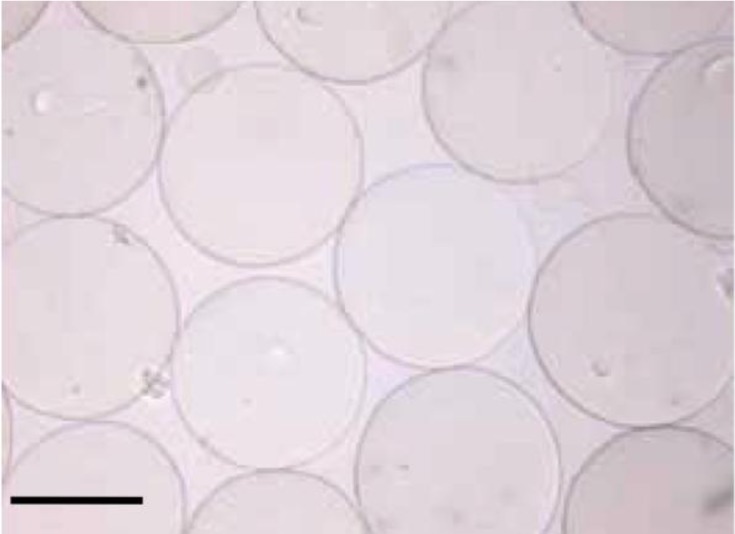
Alginate-poly(ethylene glycol) hybrid microspheres (alg-PEG-M) with an average diameter of 550 μm ± 5% SD. Scale bar: 400 μm.

**Scheme 1.**
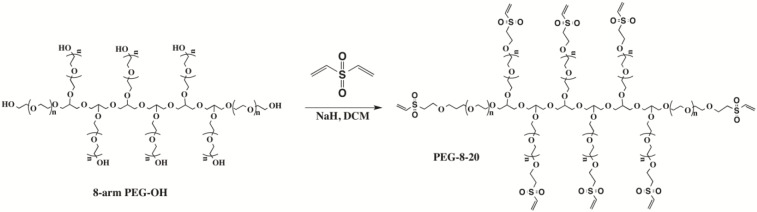
Chemical structures of 8-arm poly(ethylene glycol) (8-arm PEG-OH) and vinyl sulfone-terminated 8-arm PEG (PEG-8-20) obtained after modification of PEG-OH with a molar mass of 20 kg/mol.

Alg-PEG-M were prepared in one step by extruding the polymer solution containing PEG-8-20 (Scheme 1) and Na-alg into the gelation bath containing CaCl_2_ and the crosslinker. Physiological conditions were applied as described in detail in paragraph 3.4 (DMEM, pH = 7.4, *T* = 37 °C, osmolality ≈ 300 mOsm/kg). The microsphere diameter could be tuned by varying process conditions such as airflow, extrusion rate, and the syringe needle diameter. As a typical example, using an inner needle diameter of 400 μm, microspheres with an average diameter of 550 μm and less than 5% relative standard deviation of the diameter were obtained (Figure 1).

**Figure 2.**
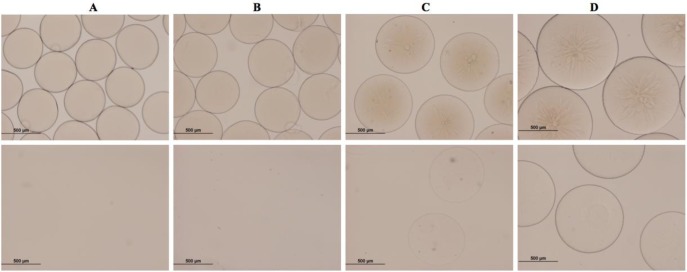
Microsphere preparations with different concentrations of vinyl sulfone-terminated multi-arm poly(ethylene glycol) (PEG-VS) in 1.5% (w/v) sodium alginate (Na-alg) PEG-VS concentrations: (A) 0% (w/v); (B) 1.25% (w/v); (C) 5% (w/v) and (D) 10% (w/v). Upper row before liquefaction; lower row same batches after liquefaction with 200 mM Na-citrate for 3 days. Scale bars: 500 μm.

The efficiency of PEG crosslinking for the here used protocol was confirmed after liquefaction of the Ca-alg hydrogel by sodium citrate, yielding stable PEG hydrogel beads above a PEG-VS concentration of 5% (*w*/*v*). Figure 2 shows stable microspheres for pure Ca-alginate beads and PEG-containing microspheres before liquefaction. Complete dissolution was observed for 1.25% (*w*/*v*) PEG-VS upon liquefaction. Figure 2 further shows that the microsphere diameter increases with increasing PEG-VS concentration when using the same encapsulation settings for all batches. Consequently, for a targeted microsphere size, the airflow and extrusion rate have to be adapted to the viscosity of the polymer solution. For all experiments and tests performed in this study, liquefaction was omitted in order to keep the microsphere production process simple. However, liquefaction remains an option for further adaptation to specific cell microencapsulation applications.

**Figure 3.**
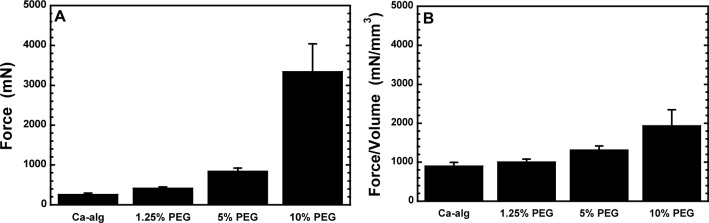
Mechanical resistance to compression up to 90% of the microsphere diameter of individual alg-PEG-M prepared with 0%–10% (*w*/*v*) PEG-VS (*n* = 30 ± SD).

The mechanical resistance to compression clearly depends on the PEG-VS concentration, as shown in Figure 3. Figure 3A presents the resistance to coaxial compression up to 90% of the diameter of the microspheres, prepared with different PEG-VS concentrations. Considering the different size of the spheres in the four batches, Figure 3B shows the volume corrected data to obtain a better comparison.

### 2.2. Microencapsulation of Human Foreskin Fibroblasts

**Figure 4.**
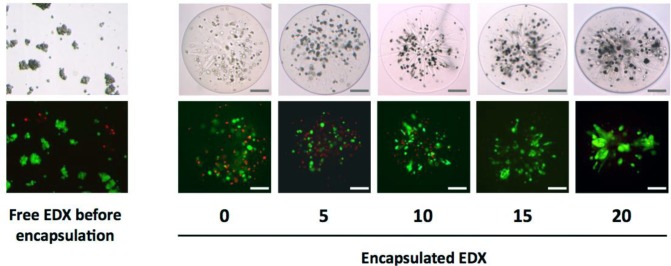
Microphotographs of non-encapsulated (left panel) and microencapsulated primary human foreskin fibroblasts (EDX cells) (right panel), visualization of representative examples at different time points (in days) up to 20 days after microencapsulation. Top, light microscopy; bottom, viability staining of the same objects with fluorescein diacetate (green: living cells) and propidium iodide (red: dead cells). The average diameter of the microspheres was 550 μm. Scale bars: 100 μm.

EDX cells were successfully microencapsulated within alg-PEG-M prepared from PEG-8-20 at 10% (*w*/*v*) in DMEM containing 1.5% (*w*/*v*) Na-alg. As shown in Figure 4, good sphericity was achieved for alg-PEG-M containing cells. The optical inspection of the gelation bath did not identify any free EDX cells. Thus, there was no out-diffusion of the cells from the polymer solution drops during the gelation process. This observation confirms that the total amount of EDX cells was embedded within alg-PEG-M. Moreover, the cells were almost homogenously distributed within the microsphere, and no empty microspheres were observed microscopically. Figure 4 shows a slight tendency of radial cell orientation, but no protruding cells were identified during the study.

### 2.3. Viability and Proliferation of Human Foreskin Fibroblasts

**Figure 5.**
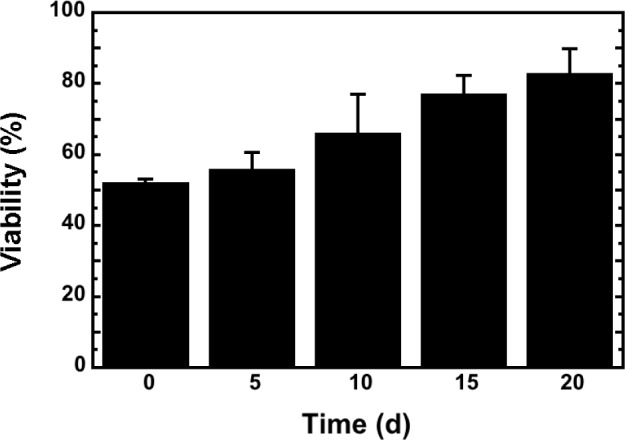
Viability of microencapsulated EDX cells at various time points (days) up to 20 days after microencapsulation.

The utilization of Dulbecco’s Modified Eagle Medium (DMEM) as medium for cell microencapsulation was proven efficient in terms of providing a cell-friendly environment during the hydrogel formation. The cell viability, assessed qualitatively by staining with fluorescein diacetate and propidium iodide, revealed good survival immediately after the microencapsulation process. Viability of free EDX cells was 80.7% ± 3.3% (mean ± SD) following trypsinization, but 51.9% ± 1.2% immediately after microencapsulation (*p* = 0.0002). However, microencapsulated EDX cells returned progressively to normal viability levels (*i.e.*, 82.7% ± 7.1% at day 20) when cultured in standard medium (IMDM supplemented with 10% fetal calf serum), *i.e.*, similar values as observed for free EDX cells (*p* = 0.6575) (Figure 5).

**Figure 6.**
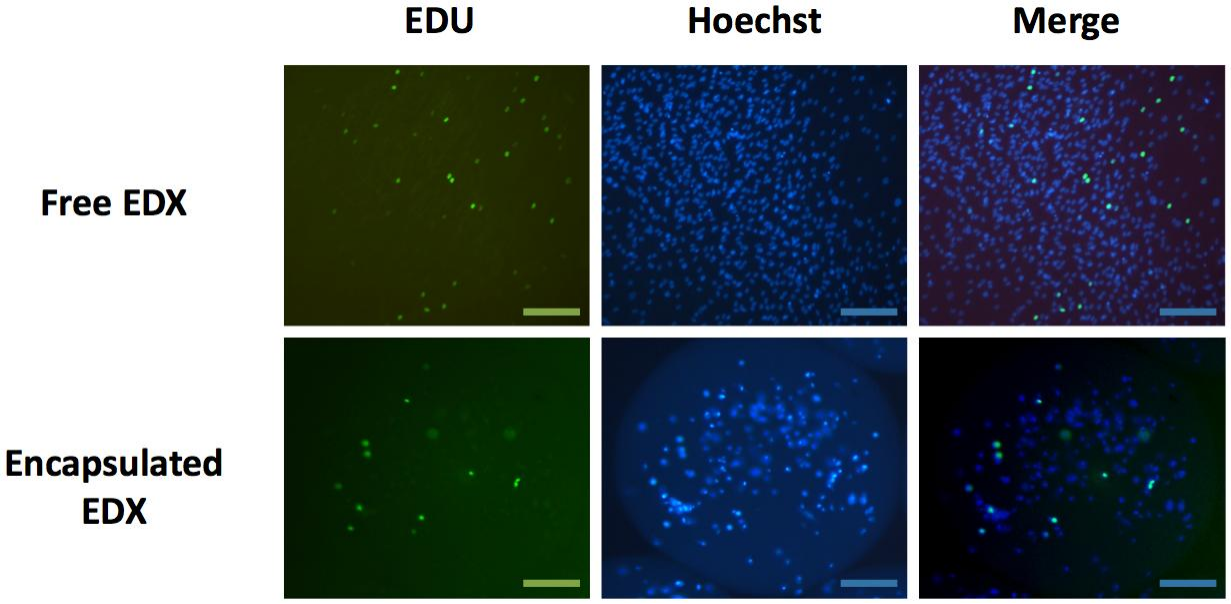
Proliferation of EDX cells 24 h after microencapsulation in alg-PEG-M. From left to right: green (EdU) shows proliferating cells; blue (Hoechst) shows all cells; Merge shows the overlay of EDU and Hoechst. Scale bars: 100 μm.

The proliferation rate, after 24 h incubation with the thymidine analog EdU, was similar for free EDX cells (6.2% ± 2.2%) and microencapsulated EDX cells (7.3% ± 4.4%, *p* = 0.6724) (Figure 6).

Human foreskin fibroblasts are primary cells of the mesenchymal lineage. They have been investigated in the field of regenerative medicine. Clinically, they have been used for the treatment of burns [32] and chronic venous ulcers [33,34]. *In vitro* experiments have shown that human foreskin fibroblasts have the potential to inhibit allogeneic mixed lymphocyte reactions and T-lymphocyte proliferation after mitogenic stimulation *in vitro* [35]. Human foreskin fibroblast cells are also often used as standard or control cells.

Using human foreskin fibroblasts allowed the demonstration that human primary cells tolerate the microencapsulation process with alg-PEG-M as hydrogel. The viability was slightly reduced immediately after the microencapsulation procedure, but returned to normal values within a few days. EDX cells recovered their normal “spindle-shaped” morphology after day 2, and kept their ability to proliferate within the microsphere at a normal rate when compared to non-encapsulated cells. The initially reduced viability upon microencapsulation can be attributed to mechanical stress applied to the cells during the extrusion through the needle. Optimized nozzle geometry could overcome this effect.

Relying on basic studies related to the range of adaptability of the permeability and mechanical properties of the alg-PEG-M two-component hydrogel, the preparation with PEG-8-20 at 10% (*w*/*v*) was selected. As previously demonstrated, using the lower molar mass PEG-8-10 yields denser polymer networks, while the opposite effect was observed for the higher molar mass PEG-8-40 [31]. Less permeable hydrogels are also obtainable by increasing the PEG concentration to values up to 20% (*w*/*v*). However, there was a tendency that too compact hydrogels lead to inhomogeneous microsphere structures with a more compact core surrounded by a more permeable layer. The selection of 8-arm PEG of the middle molar mass of 20 kg/mol and preparation at 10% (*w*/*v*) in 1.5% (*w*/*v*) Na-alg was confirmed as a suitable protocol for the microencapsulation of EDX cells. Inhomogeneity or phase separation were not observed, neither immediately after the preparation nor during storage in cell culture medium with serum supplements. Moreover, the microsphere size remained constant upon changing the culture medium during the experiment.

We conclude that this study is an encouraging step towards transplantation of allo- and xeno-geneic microencapsulated cells in order to treat congenital or acquired hormone/enzyme deficiencies as well as degenerative/inflammatory diseases.

## 3. Experimental Section

### 3.1. Reagents

Na-alg (PRONOVA UP LVM) was obtained from FMC BioPolymer (Novamatrix, Drammen, Norway, batch no FP-506-01). 8-arm PEG, molar mass 20 kg/mol, was purchased from JenKem (JenKem Technology USA Inc, Allen, TX, USA). This PEG consists of a poly(glycerol) backbone with multiple PEG arms attached through an ether bond (PEG-OH) (Scheme 1). Dulbecco’s Modified Eagle Medium (DMEM, special formulation without NaCl and KCl) was purchased from Cell Culture Technologies LLC (Gravessano, Switzerland). Divinylsulfone, DL-dithiothreitol (DTT), calcium chloride dihydrate, and sodium chloride were obtained from Sigma (Sigma-Aldrich, Buchs, Switzerland). All chemicals were of analytical grade and were used as supplied, unless otherwise indicated.

### 3.2. Analytical Methods and Instrumentation

Microphotographs were taken with an Olympus AX70 microscope connected to an Olympus DP70 color digital camera. The image analysis was performed using Olympus DP Manager software (Olympus, UK). The osmolality of the solutions was measured using a Micro-sample Osmometer (Fiske^®^, 210, Noorwood, MA, USA). Mechanical resistance measurements were performed at room temperature using a Texture Analyzer TA.XT.Plus (Stable Micro System LDT, Godalming, Surrey, UK) equipped with Texture Exponent 32 software (Stable Micro System LDT) for data analysis.

### 3.3. Functionalization of Poly(ethylene glycol) with Vinyl Sulfone End Groups

The vinyl sulfone-terminated derivative of PEG-OH (Scheme I) was synthesized as described previously [30]. The functionalized 8-arm PEG having the molar mass of 20 kg/mol is designated as PEG-8-20.

### 3.4. Formation of Microspheres

All components necessary for the preparation of microspheres were dissolved in a special DMEM preparation, which contained neither NaCl nor KCl in order to avoid too high osmolality upon dissolution of the polymers and salts. After dissolution of the reagents at their desired concentration, the osmolality was adjusted to that of basic DMEM without supplements (≈300 mOsm/kg) by adding NaCl. The gelation bath was prepared by dissolving CaCl_2_ and DTT, in DMEM and adjusting the osmolality to 300 mOs/kg (80 ± 5 mM CaCl_2_). The microspheres were prepared under sterile conditions employing a coaxial airflow droplet generator [36].

As a typical example, 1.1 g of PEG-8-20 was dissolved in 10 mL of 1.65% (*w*/*v*) Na-alg aqueous stock solution yielding a solution, which had the final concentrations of 1.5% (*w*/*v*) Na-alg and 10% (*w*/*v*) PEG-VS. The solution was then sterile filtered (0.2 μm), and 10 mL was extruded into 100 mL of the gelation bath containing 85 mM Ca^2+^ and 85 mg DTT. The receiving bath was incubated in a shaker (80 rpm) at 37 °C for up to 3 h to achieve optimal crosslinking [30]. Alg-PEG-M were collected by filtration.

For cell microencapsulation, the cells were added to the polymer solution before the extrusion into the gelation bath. After gelation and separation, the cell-containing microspheres were stored in the cell culture medium.

### 3.5. Mechanical Testing

Single microspheres were placed below the Texture Analyzer probe, which traveled toward the lower plane with a constant speed set at 0.5 mm/s. During probe displacement, the resistance of the sample to the compression was recorded up to a compression of 98% of the initial microsphere diameter. Thirty spheres of each batch were individually analyzed. The average mechanical resistance was then expressed as the mean ± standard deviation (SD).

### 3.6. Microencapsulation of Primary Human Foreskin Fibroblasts

Primary human foreskin fibroblasts (in this study designated as EDX cells, a gift from DFB Bioscience) were detached using 0.25% trypsin-EDTA (Sigma-Aldrich, Buchs, Switzerland) for about 30 s and washed twice. The EDX cell suspension was centrifuged at room temperature (1200 rpm, 5 min) and the supernatant withdrawn. The pellet was resuspended in Na-alg/PEG-8-20 solution (1.5% (*w*/*v*) Na-alg + 10% (*w*/*v*) PEG-8-20 in DMEM) to a final concentration of 500,000 cells/mL. The mixture was gently homogenized using a pipette and subsequently extruded into the sterile receiving bath, as described in Section 3.4. After filtration and washing, free and microencapsulated EDX cells were cultured in Iscove’s modified Dulbecco’s Medium (IMDM) (Cambrex, Verviers, Belgium) supplemented with 10% fetal calf serum (Gibco-Invitrogen, Basel, Switzerland), 100 IU/mL penicillin, and 100 mg/mL streptomycin (Gibco-Invitrogen). The medium was changed every three days.

### 3.7. Viability and Proliferation Assays

Fluorescein diacetate (FDA) living cell staining and propidium iodide (PI) dead cell staining (both from Sigma) were used to assess EDX cell viability. A mixture with FDA or PI was incubated for 2 min prior to evaluation. Cell viability was assessed immediately after microencapsulation (day 0) and at days 5, 10, 15, and 20. 5000 free and 1000 microencapsulated EDX cells were analyzed. The ratio between FDA-positive and PI-positive EDX cells was calculated using offline MetaMorph imaging software for microscopy (Universal Imaging, West Chester, PA, USA). To analyze proliferation, 5-ethynyl-2’-deoxyuridine (EdU), a nucleoside analog of thymidine that is incorporated into DNA during active DNA synthesis, was added to the culture medium immediately after microencapsulation. After 24 h, free or encapsulated EDX cells were fixed with 4% paraformaldehyde for 15 min and permeabilized using 0.5% Triton X-100 for 5 min. Proliferating cells were detected using the histochemical assay kit as described by the manufacturer (Click-iT^®^ EdU Cell Proliferation Assays, Invitrogen Corp., Carlsbad, CA, USA). Hoechst 33342 (Sigma) was used to stain all EDX cell nuclei. A total of 4000 free and 1000 microencapsulated EDX cells were analyzed. Proliferation was expressed as percentage of proliferating cells (EdU-positive) with respect to the total number of cells (Hoechst-positive), using offline MetaMorph imaging software.

### 3.8. Statistical Analysis

The results were expressed as the mean ± standard deviation. Unpaired Student’s *t*-test was used to compare the mean values. A two-sided p value <0.05 was considered significant. Computations were performed using GraphPad Prism, version 4.0 (GraphPad Software, Inc., La Jolla, CA, USA).

## 4. Conclusions

The development of novel materials and microencapsulation procedures contributes to accelerating the clinical implementation of cell microencapsulation. Moreover, from recent studies it can be concluded that the presence of polycations in the microspheres as well as multi-step encapsulation processes have disadvantages. Considering these aspects, novel hydrogel microspheres free of polycations and produced in one step in cell culture media have been developed and tested in the present study.

Alg-PEG-M microspheres were prepared by a process, which takes place in cell culture medium. Such a medium was used for both polymer solvent and solution with physiological osmolality to promote cell survival and integrity. The use of cell culture media neither negatively affected the preparation nor the physical properties of this type of hybrid microsphere. In addition, there are no positive charges present in the hydrogel, which could induce and promote protein and cell attachment.

Microencapsulation of primary cells, herein human foreskin fibroblasts, resulted in promising data regarding cell survival and function. The cell viability immediately after the microencapsulation procedure was slightly lower than for free cells. There was a similar proliferation rate observed for cells inside the microspheres and for free cells.

This first set of experiments confirmed the feasibility and physiological compatibility of cell microencapsulation within alg-PEG-M. This study is considered fundamental for future adaptation of alg-PEG-M to specific cell microencapsulation applications.

## References

[1] Bonavita A.G., Quaresma K., Cotta-de-Almeida V., Pinto M.A., Saraiva R.M., Alves L.A (2010). Hepatocyte xenotransplantation for treating liver disease. Xenotransplantation.

[2] Paul A., Ge Y., Prakash S., Shum-Tim D (2009). Microencapsulated stem cells for tissue repairing: Implications in cell-based myocardial therapy. Regener. Med.

[3] De Vos P., Faas M.M., Strand B., Calafiore R (2006). Alginate-based microcapsules for immunoisolation of pancreatic islets. Biomaterials.

[4] Hernández R.M., Orive G., Murara A., Pedraz J.L. (2010). Microcapsules and microcarriers for *in situ* cell delivery. Adv. Drug Deliv. Rev.

[5] Rokstad A.M., Brekke O.L., Steinkjer B., Ryan L., Kolláriková G., Strand B.L., Skjåk-Braek G., Lacik I., Espevik T., Mollnes T.E. (2011). Alginate microbeads are complement compatible, in contrast to polycation containing microcapsules, as revealed in a human whole blood model. Acta Biomater.

[6] Rokstad A.M., Brekke O.L., Steinkjer B., Ryan L., Kolláriková G., Strand B.L., Skjåk-Braek G., Lambris J.D., Lacik I., Mollnes T.E. (2013). The induction of cytokines by polycation containing microspheres by a complement dependent mechanism. Biomaterials.

[7] Moyer H.R., Kinney R.C., Singh K.A., Williams J.K., Schwartz Z., Boyan B.D. (2010). Alginate microencapsulation technology for the percutaneous delivery of adipose-derived stem cells. Ann. Plast. Surg.

[8] Malpique R., Osorio L.M., Ferreira D.S., Ehrhart F., Brito C., Zimmermann H., Alves P.M. (2010). Alginate encapsulation as a novel strategy for the cryopreservation of neurospheres. Tissue Eng. Methods.

[9] Park H.S., Ham D.S., You Y.H., Shin J., Kim J.W, Jo J.H, Kim O.Y., Khang G., Yoon K.H (2010). Successful xenogenic islet transplantation with Ba^2+^-Alginate encapsulation. Tissue Eng. Regen. Med.

[10] Penolazzi L., Tavanti E., Vecchiatini R., Lambertini E., Vesce F., Gambari R., Mazzitelli S., Mancuso F., Luca G., Nastruzzi C. (2010). Encapsulation of mesenchymal stem cells from Wharton’s Jelly in alginate microbeads. Tissue Eng. Methods.

[11] Endres M., Wenda N., Woehlecke H., Neumann K., Ringe J., Erggelet C., Lerche D., Kaps C (2010). Microencapsulation and chondrogenic differentiation of human mesenchymal progenitor cells from subchondral bone marrow in Ca-alginate for cell injection. Acta Biomater.

[12] Cui H., Tucker-Burden C., Cauffield S.M.D., Barry A.K., Iwakoshi N.N., Weber C.J., Safley S.A. (2009). Long-term metabolic control of autoimmune diabetes in spontaneously diabetic nonobese diabetic mice by nonvascularized microencapsulated adult porcine islets. Transplantation.

[13] Dang T.T., Thai A.V., Cohen J., Slosberg J.E., Siniakowicz K., Doloff J.C., Ma M., Hollister-Lock J., Tang K.M., Gu Z. (2013). Enhanced function of immuno-isolated islets in diabetes therapy by co-encapsulation with an anti-inflammatory drug. Biomaterials.

[14] Giovagnoli S., Blasi P., Luca G., Fallarino F., Calvitti M., Mancuso F., Ricci M., Basta G., Becchetti E., Rossi C. (2010). Bioactive long-term release from biodegradable microspheres preserves implanted ALG-PLO-ALG microcapsules from *in vivo* response to purified alginate. Pharm. Res.

[15] De Castro M., Orive G., Hernández R.M., Bartkowiak A., Brylak W., Pedraz J.L. (2009). Biocompatibility and *in vivo* evaluation of oligochitosans as cationic modifiers of alginate/Ca microcapsules. J. Biomed. Mater. Res.

[16] Babister C., Tare R.S., Green D.W., Inglis S., Mann S (2008). Genetic manipulation of humanmesenchymal progenitors to promote chondrogenesis using “bead-in-bead” polysaccharide capsules. Biomaterials.

[17] Baruch L., Machluf M (2006). Alginate-chitosan complex coacervation for cell encapsulation: Effect on mechanical properties and on long-term viability. Biopolymers.

[18] Yu C.B., Lv G.L., Pan X.P., Chen Y.S., Cao H.C., Zhang Y.M., Du W.B., Yang S.G., Li L.J. (2009). *In vitro* large-scale cultivation and evaluation of microencapsulated immortalized human hepatocytes (HepLL) in roller bottles. Int. J. Artif. Organs.

[19] Luna S.M., Gomes M.E., Mano J.F., Reis R.L. (2010). Development of a novel cell encapsulation system based on natural origin polymers for tissue engineering applications. J. Bioact. Compat. Pol.

[20] Mazumder M.A.J., Burke N.A.D., Shen F., Potter M.A., Stöver H.D.H. (2009). Core crosslinked alginate microcapsules for cell encapsulation. Biomacromolecules.

[21] Gardner C.M., Burke N.A.D, Stöver H.D.H. (2010). Cross-linked microcapsules formed from self-deactivating reactive polyelectrolytes. Langmuir.

[22] Gardner C.M., Potter M.A., Stöver H.D.H. (2012). Improving covalent cell encapsulation with temporarily reactive polyelectrolytes. J. Mater. Sci. Mater. Med.

[23] Rokstad A.M., Brekke O.L., Steinkjer B., Ryan L., Kolláriková G., Lambris J.D., Lacik I., Mollnes T.E., Espevik T (2012). Poly-cation containing alginate microcapsules induce cytokines by a complement-dependent mechanism. Immunobiology.

[24] Luan N.M., Teramura Y., Iwata H (2010). Immobilization of the soluble domain of human complement receptor 1 on agarose-encapsulated islets for the prevention of complement activation. Biomaterials.

[25] Stiegler P., Matzi V., Pierer E., Hauser O., Schaffellner S., Renner H., Greilberger J., Aigner R., Maier A., Lackner C. (2010). Creation of a prevascularized site for cell transplantation in rats. Xenotransplantation.

[26] Wells L.A., Sheardown H (2011). Photosensitive controlled release with polyethylene glycol-anthracene modified alginate. Eur. J. Pharm. Biopharm.

[27] Davidovich-Pinhas M., Bianco-Peled H (2011). Physical and structural characteristics of acrylatedpoly(ethylene glycol)–alginate conjugates. Acta Biomater.

[28] Yang J.S., Xie Y.J., He W (2011). Research progress on chemical modification of alginate: A review. Carbohydr. Polym.

[29] Hall K.K., Gattás-Asfura K.M., Stabler C.L. (2011). Microencapsulation of islets within alginate/poly(ethylene glycol) gels cross-linked via Staudinger ligation. Acta Biomater.

[30] Mahou R., Wandrey C (2010). Alginate-poly(ethylene glycol) hybrid microspheres with adjustable physical properties. Macromolecules.

[31] Mahou R., Kolláriková G., Gonelle-Gispert C., Meier R., Schmitt F., Tran N.M., Dufresne M., Lacik I., Bühler L., Juillerat-Jeanneret L. (2013). Combined electrostatic and covalent polymer networks for cell microencapsulation. Macromol. Symp.

[32] Nanchahal J., Dover R., Otto W.R. (2002). Allogeneic skin substitutes applied to burns patients. Burns.

[33] Falanga V., Margolis D., Alvarez O., Auletta M., Maggiacomo F., Altman M., Jensen J., Sabolinski M., Hardin-Young J (1998). Rapid healing of venous ulcers and lack of clinical rejection with an allogeneic cultured human skin equivalent. Arch. Dermatol.

[34] Yonezawa M., Tanizaki H., Inoguchi N., Ishida M., Katoh M., Tachibana T., Miyachi Y., Kubo K., Kuroyanagi Y (2007). Clinical study with allogeneic cultured dermal substitutes for chronic leg ulcers. Int. J. Dermatol.

[35] Wada N., Bartold P.M., Gronthos S (2011). Human foreskin fibroblasts exert immunomodulatory properties by a different mechanism to bone marrow mesenchymal stem cells. Stem Cells Dev.

[36] Ceausoglu I., Hunkeler D (2002). A new microencapsulation device for controlled membrane and capsule size distributions. J. Microencapsul.

